# Impact of grazing around industrial areas on milk heavy metals contamination and reproductive ovarian hormones of she-camel with assessment of some technological processes on reduction of toxic residue concentrations

**DOI:** 10.1186/s12917-024-03882-7

**Published:** 2024-01-31

**Authors:** Asem Mohammed Zakaria, Yahia A. Amin, Haydi Mohamed Zakaria, Foad Farrag, Liana Fericean, Ioan Banatean-Dunea, Mohamed Abdo, Ahmed Hafez, Ragab Hassan Mohamed

**Affiliations:** 1 Department of Food Hygiene, Faculty of Veterinary Medicine, Aswan University, Aswan, Egypt; 2 Department of Theriogenology, Faculty of Veterinary Medicine, Aswan University, Aswan, Egypt; 3 Department of Clinical Research and Health Development, Menoufia Directorate of Health Affairs, Ministry of Health and population, 32511 Shebin El-Kom, Menoufia, Egypt; 4 Department of Anatomy and Embryology, Faculty of Veterinary medicine, Kafr-elsheikh University, Kafr-elsheikh, Egypt; 5 Department of Basic Veterinary Sciences, Faculty of Veterinary Medicine, Delta University for Science and Technology, 7730103 Dakahlia, Egypt; 6 Department of Biology and Plant Protection, Faculty of Agricultural Sciences, University of Life Sciences King Michael I, 300645 Timisoara, Romania; 7 Department of Animal histology and anatomy, school of Veterinary Medicine, Badr University in Cairo (BUC), Cairo, Egypt; 8 Department of Anatomy and Embryology, Faculty of Veterinary Medicine, University of Sadat City, Sadat city, Egypt; 9 Department of Pharmacology, Faculty of veterinary medicine, Aswan University, Aswan, Egypt

**Keywords:** Heavy metals, Milk, Ovarian hormones, She-camel, Technological processes

## Abstract

Heavy metals are one of the most toxic chemical pollutants of the environment. Their hazards not restricted to human but extend to animal productivity and reproductively. The present study aimed to assess the impact of grazing around industrial areas on the levels of copper (Cu) and aluminum (Al) residues in milk samples collected from dromedary she-camels and studying their effects on some ovarian hormones. In addition, the study aimed to investigate methods of removal of the toxic concentrations of these heavy metals in milk by applying different technological processes. Blood and milk samples were collected from 30 dromedary she-camels, 15 grazing in non-industrial areas (group A) and 15 grazing in industrial areas (group B). Detection of the levels of these heavy metals in milk was done. Ovarian hormones investigation on the blood was performed. Different technological processes such as boiling, skimming and fermentation were applied to all contaminated samples to reduce the toxic concentrations of these heavy metals. Results revealed that all examined milk samples in both groups contained Cu, while 40% of group A and 100 % of group B contained Al residues with different concentrations. The levels of Cu and Al residues in samples of group A not exceeded the maximum residual limit (MRL) set by World Health Organization (WHO) while 60% and 100% of milk samples in group B contained Cu and Al residues exceeded MRL, respectively. Technological processes induce variant changes in the levels of these metals in milk. Heat treatment of milk in Al vats leads to leaching of Al from containers to the milk causing significant increase in Al load, while Cu level was not significantly affected. Boiling in stainless-steel containers decreased the levels of Al and Cu but in non-significant levels. Regarding skimming process, small amount of Cu and Al escaped into the skimmed milk while greater amount were recovered in the cream. Fermentation by probiotic bacteria showed that milk fermentation has non-significant effect on Cu and Al levels. Investigation of ovarian hormones (estrogen and progesterone) revealed presence of a signification reduction in the levels of these hormones in group B compared to group A. In addition, a negative correlation was found between these heavy metals and ovarian hormones concentrations in the blood. It is concluded that grazing of dromedary camels around industrial areas induce heavy metals toxicity represented by excretion of these metals in milk and significant reduction on ovarian function showed by reduction of estrogen and progesterone levels. Technological processes such as skimming decreased the levels of Al and Cu residues in milk.

## Introduction

Numerous dairy animals, including buffalo, cattle, goats, sheep, and camels, are utilized as the main supply of milk [1]. Because of their inherent qualities and the health advantages of their milk, camels are regarded as one of the most significant animals [2]. Camel milk contains high protective proteins as lactoperoxidase, lysozyme, lactoferrin, and immuno-globulins, high minerals as magnesium, iron, sodium, potassium, copper and zinc, high vitamin C, low cholesterol and low sugar [3]. It has been used for treatment of many diseases as jaundice, asthma, anti-hypertensive, leishmaniasis and dropsy [4].Milk is not only a source of nutrition, but its production also contributes to food security and income for most people in the developing countries. Around 150 million households are engaged in milk production across the globe [5]. It is particularly beneficial for small scale producers because of quick cash turnouts. Due to all these health benefits, nutritional values and economic importance, awareness was increased and attention was given to study various factors affecting safety and quality of camel milk [6].

There are several safety risks associated with milk that originate from exposure of milk to chemicals, microbial contamination, illegal additives, aflatoxins [7–9], veterinary drugs [10, 11] and heavy metals [12]. Heavy metals are one of the most dangerous environmental pollutants that contaminate the milk creating significant risk to consumers [13]. Heavy metals are found in the environment naturally or released by anthropogenic activities such as burning of the organic matter, mining, or direct disposal of the industrial and agricultural wastes [14]. They contaminate milk directly through contaminated air, water, milk utensils and equipment or indirectly through animal feeds which eventually finds its way into milk [15]. Heavy metals are characterized by their bio-accumulative and biomagnification nature causing many cases of human and animal body intoxication and multiple organ damage [16].

The presence of toxic metals in milk and meat, some of which are hazardous to humans, is a major issue for food safety [17]. Lead (Pb), mercury (Hg), cadmium (Cd), arsenic (As), cobalt (Co), chromium (Cr), lithium (Li), molybdenum (Mo), aluminum (Al), and copper (Cu) are elements that are categorized as hazardous metals [16, 18].

Aluminium (Al) is the third most abundant element in the environment, comprising 8.13% of the earth’s crust [19]. It is present naturally in rocks, minerals and soil even in food and water. Al doesn’t have any physiological or biological values in the human body [20].

Recent investigations on environmental toxicology revealed that Al may present a major threat for humans, animals and plants in causing many diseases as osteomalacia, anemia, serious brain disorders like Alzheimer’s and dialysis encephalopathy especially in chronic renal failure patient [21].

Copper (Cu) is one of the essential microelements for human body. It acts as a cofactor of some body enzymes that are essential for different vital processes in the body, required for the iron absorption, essential in blood clotting and distribution of stimuli in the nervous system [22]. Cu deficiency may cause anemia, heart and vascular diseases and decrease in body growth and development. However, its higher intake above the maximum permissible limit (0.1ppm) recommended by the different international standards may cause adverse effects to human health as kidney and liver damage, anemia, bowel and stomach irritation [15]. The main sources of Cu and Al in the diet are milk, milk products beverages, desserts and cereals [23].

Heavy metals also have a harmful effect on the reproductive system. Long exposure to heavy metals leads to chronic toxicity and produce functional and structural cellular impairments. Toxic metals concentrated in the follicular fluid of the female reproductive system and harm the ovarian granulosa cells, which impairs hormone synthesis and lowers the quality of oocytes [24]. Toxic metals can also result in premature calving or apportion. Additionally, they have the ability to pass through the placenta and harm the developing fetus [18, 25].

Toxic metals in the male reproductive system disrupt spermatogenesis, induce sperm apoptosis, cause oxidative damage, and may contribute to male infertility [18, 26]. Heavy metals accumulate mainly in the seminal vesicles, epididymis, testes, vas deferens, and semen [26]. Bulls exposed to heavy metals had lower quality semen and less sperm count [26]. Furthermore, animals exposed to heavy metals usually suffer from hemorrhage, necrosis, or loss of germ cells in calves [26].

The current study hypothesis that during grazing dromedary she camels around industrial areas they are exposed to heavy metals or toxic chemical compounds which enter into the animals’ body through the respiratory and digestive systems and end up in their milk.

Therefore, In the present study we aimed to monitor the level of heavy metals (Cu and Al) residues in milk samples collected from dromedary she camels that grazing in some industrial areas to determine the extent of milk contamination, health risk assessment and investigate their effect on some reproductive hormones. In addition, several technological processes are applied in trial to remove or reduce the levels of these heavy metals in milk.

## Materials and methods

### Animals

Animals were privately owned by individuals living in the area of study.

**Fig. 1:**
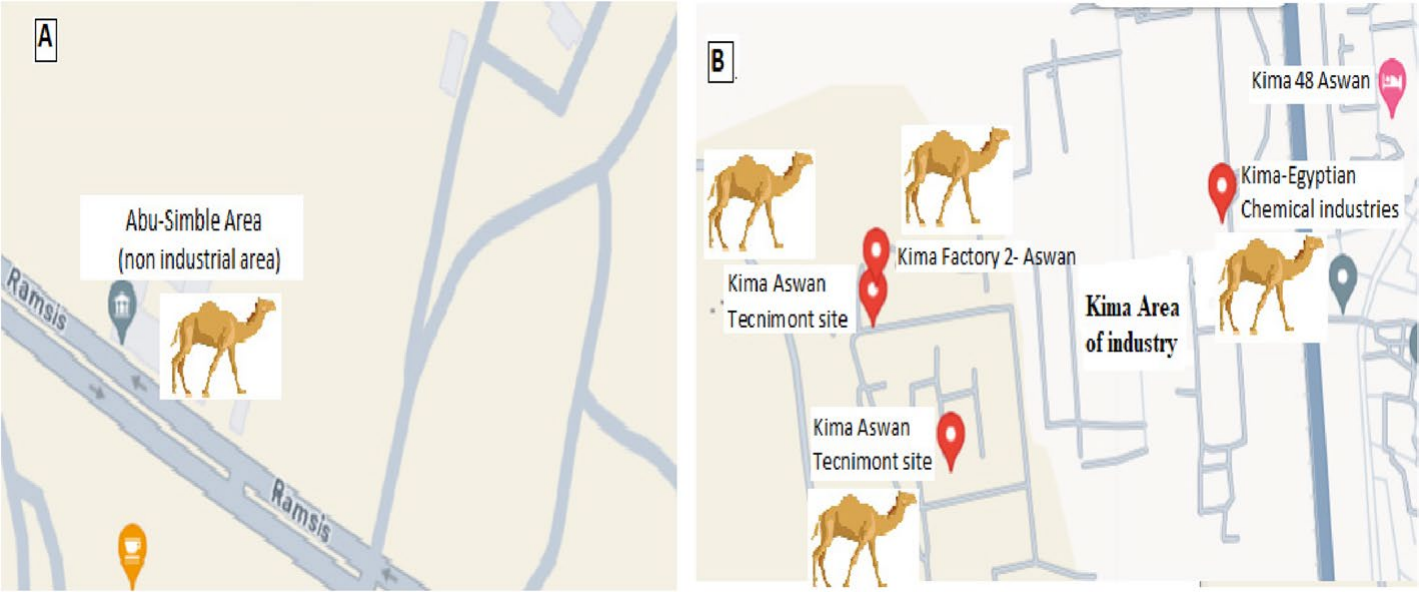
Map depicting the study area in Aswan Province, Egypt. The locations of the sampling sites are indicated and including the industrial site in KIMA’s area of industry (**B**), Non industrial area of Abu-Simble (**A**), located 285 km away from KIMA’s industrial site

The experiment was conducted during the period from December 2021 to April 2023. Thirty pluriparous, cyclic, lactating and apparently healthy she-camels with a body weight of 500 ± 30 kg and age of 6-14 years old were included (15 grazing in non-industrial areas (group A) and 15 grazing in industrial areas (group B) ) in the South of Egypt (Fig. 1). At the beginning of the experiment, all she-camels were exhibited clinically healthy. The camels were considered clinically healthy on the basis of physical examination of heart rate, lungs, rumen, intestine, normal body temperature, respiration, feed intake and normal movement of the animal [2].

KIMA’s factories were established at the South of Aswan on an area of 1500 feddans. These factories are specialized in producing nitro‐kima fertilizer, pure ammonium nitrates, aluminum, ferrosilicon nitrates and fertilizers. KIMA were taken as example for some industrial areas present in south Egypt.

### Milk samples

Thirty Milk samples (500 ml each) were collected individually from 30 hand milked dromedary she-camel in screw-capped bottles. All samples transferred to laboratory in ice box and kept frozen at -20°C till be examined.

### Blood sampling and hormonal analysis

Thirty blood samples (10 ml each) were collected from each she-camel separately via jugular vein puncture using vacutainer tubes without anticoagulant to obtain serum [27]. Serum samples were analyzed for determination of ovarian hormones level. Hormonal analyses were done using Ref: EA 74 and KGE014 commercial test kits for progesterone and estradiol, respectively, supplied by Oxford Biomedical Research USA. All kits were used according to the standard protocols of suppliers.

### Estimation of Cu and Al concentrations in milk samples

Concentration of Cu and Al were determined by Inductivity Coupled Argon Plasma, iCAP6500 Duo, Thermo Scientific, England [28]. 1000mg/l multi-element certified standard solution, Merck, Germany was used as a stock solution for instrument standardization.

#### Samples preparation

Prior to digestion, milk samples were defrosted overnight at 4°C. Distilled water and nitric acid 30% were used for washing all bottles and glass tubes, then air dried after washing by distilled water and kept clean tell be used. All milk samples were kept in 1:1 nitric acid for 24 hand rinsed well with double distilled water (ddH2O).

#### Digestion of milk samples and estimation of heavy metals concentration

Twenty five mL of the milk samples were digested by 7 mL of nitric acid (HNO3) and7 mL of 30% hydrogen peroxide (H2O2) in a digestion apparatus.ddH2O were used to adjust the volume of digested samples to 50 mL after cooling to room temperature. The clear filtrate of each digested sample was kept refrigerated to avoid evaporation. Blank samples were prepared in the same way. Digested samples were being analyzed for Cu and Al using CAP 6500 Duo.

### Effect of different processing treatments on heavy metals concentrations

#### Effect of milk boiling in different pans on Cu and Al concentrations

Water bath were used for milk boiling. 200 ml from each milk sample were taken and 100 ml of them were poured into aluminum pan. The another 100 ml were poured into stainless-steel pan and boiled in water bath at 100.5°C for 10 mints then reexamined by inductivity coupled argon plasma to determine the level of Cu and Al after boiling.

#### Effect of skimming on Cu and Al concentrations in milk samples

Centrifugal separator was used for skimming of milk samples. Then the skimmed milk and cream fractions were examined by inductivity coupled argon plasma to determine the effect of skimming on Cu and Al concentrations in milk.

#### Effect of fermentation on the concentration of Cu and Al in milk samples

Lyophilized starter culture (YoFlex^®^ Express 2.0Chr Hansen, Horsham, Denmark), containing *S. thermophilus and L. bulgaricus* were used in milk fermentation [29]. After fermentation the pH of fermented milk samples were measured by pH meter. Later, the samples were kept in the refrigerator at 4°C for 24 h then examined by inductivity coupled argon plasma to determine the level of Cu and Al after fermentation.

### Statistical analysis

Data were collected and analyzed using SPSS program for statistical analysis (version 21; IBM Corporation, Armonk, NY, USA). Quantitative data were shown as mean ± stander deviation (SD) (minimum-maximum). Mann-Whitney U test was used for continuous non-parametric variables between 2 groups. The Kruskal-Wallis test was used to measure significance among more than 2 quantitative variables non-parametrically distributed. Significant variables underwent multilevel comparisons using post-Hoc analysis. The results of comparing the correlation between two continuous variables were indicated by the correlation coefficient (r) using Spearman correlation analysis. P value was considered to be of statistical significance if it is ≤ 0.05.

## Results

### Detection of copper and aluminum in camel milk

Results showed that 100% and 40% of tested milk samples in the group A contained Cu and Al residues respectively. While in group B 100% of tested milk samples contained both Cu and Al residues. The Mean ± SD concentration of heavy metals residues in group B were higher than that in group A.

### Maximum residual limit (MRL) set by World Health Organization for Cu and Al residues in milk

World Health Organization (WHO) set MRL for Cu and Al residues in milk and the data reported in Table 2 revealed that concentration of Cu and Al in group A were below the MRL, while in group B, 60% and 100 % of tested milk samples contained Cu and Al residues in concentrations exceeded MRL respectively.

### Effect of different processing treatments on Cu and Al levels in milk samples

#### Effect of milk boiling in aluminum and stainless-steel pans on heavy metals concentrations

**Fig. 2:**
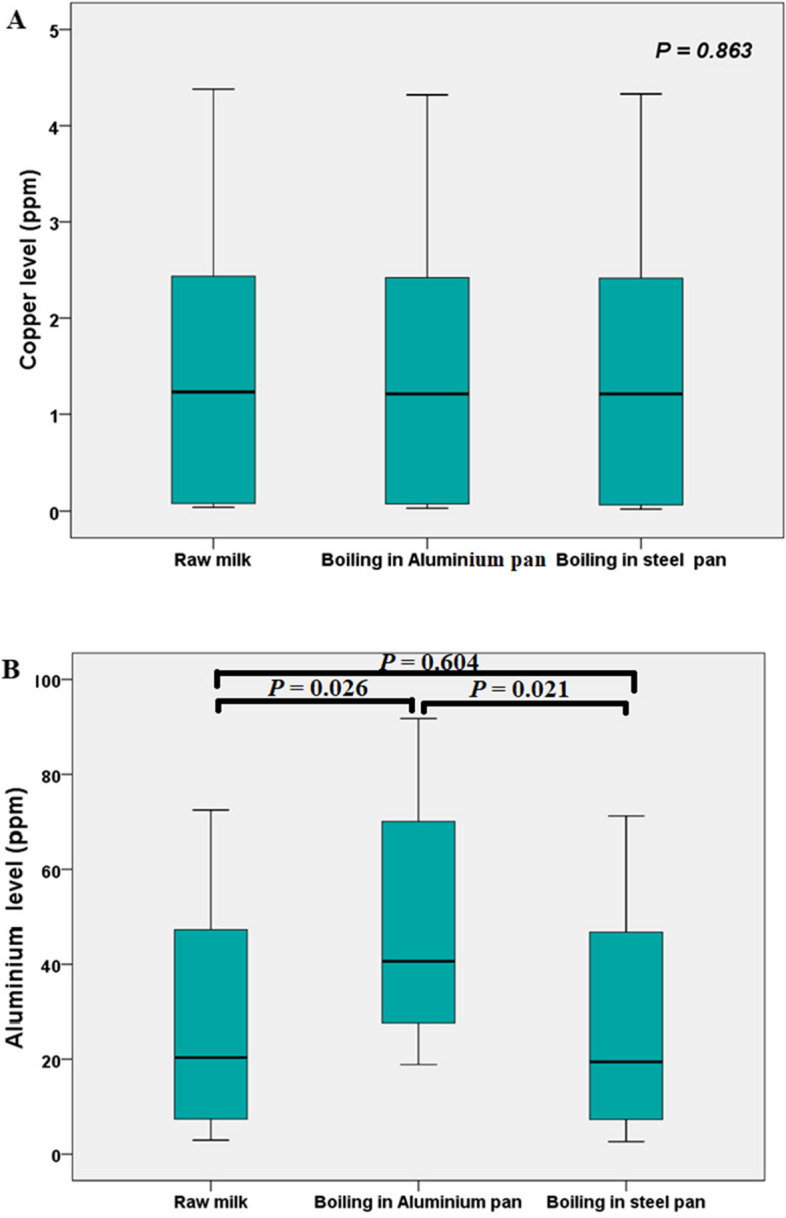
Effect of milk boiling in aluminum and stainless-steel pans on Cu and Al levels

Data reported in Fig. 2 (A and B) showed that the mean ± SD concentration of Cu in original sample was 1.43 ± 0.71 and after boiling in aluminum pan was 1.42 ± 0.62 while after boiling in stainless-steel pan was 1.41 ± 0.73ppm. On the other hand the mean ± SD concentration of Al in original sample was 27.80 ± 1.48 and after boiling in aluminum pan was 48.08 ± 1.14 while after boiling in stainless-steel pan was 27.30 ± 1.24ppm.

The upper and lower transverse lines of the box showing the 75^th^ and 25^th^ percentile respectively and the line in between showing the median. The upper and lower transverse lines of the whiskers showing the maximum and the minimum levels respectively.

#### Effect of skimming on heavy metals concentrations in milk samples

**Fig. 3:**
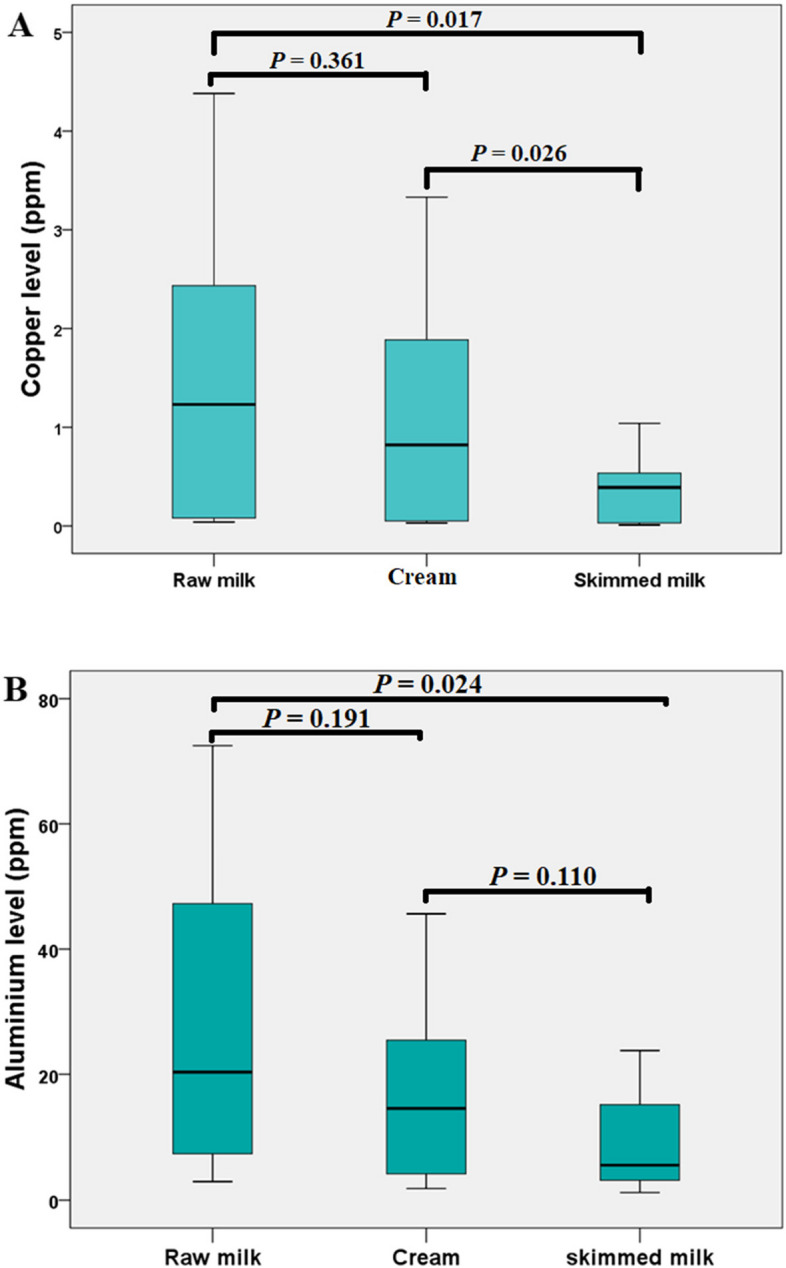
Effect of skimming on Cu and Al levels in milk samples

Data reported in Fig 3 (A and B) showed that the mean ± SD concentration of Cu and Al in original samples were 1.43 ± 0.71 and 27.80 ± 1.48 ppm respectively while after skimming were 1.09 ± 0.12 and 16.70± 1.02 ppm in fat layer and 0.33±0.02 and 9.44±0.76 ppm in skim milk portion for Cu and Al respectively.

#### Effect of fermentation on heavy metals residues in milk samples

Probiotic bacteria were used for milk fermentation and the results showed that fermentation has no significant effect (*P* > 0.05) on Cu and Al levels in the tested milk samples (Table 3).

### Relation between Cu and AL residues and ovarian hormones of female dromedary camel

#### Level of estrogen and progesterone in serum of she-camel

Results of estrogen and progesterone showed that there are a significant reduction in the levels of hormones in group B compared to group A

#### Correlation between Cu and Al concentrations and ovarian hormones concentrations in she-camel

Results showed that there was a significant negative correlation between heavy metals (Cu and Al) concentrations and ovarian hormones (estrogen and progesterone) concentrations in blood (Table 5).

## Discussion

Rabid growth of chemical and agriculture industries increased the incidence of environmental pollution with heavy metals [30]. Human and animals can be exposed to heavy metals through food chain [31]. Milk considered as one of the most important daily diets from food chain especially for children and old age peoples [32]. Milk can be used as an indicator of environmental pollution since it can become contaminated either before it is milked from a contaminated area or after it is milked during processing and storage [33]. So regular examination of milk for presence of heavy metals is necessary to insure its safety and quality.

In the present study, milk samples were examined for presence of Cu and Al residue to determine the effect of gazing around industrial areas on milk contamination. Data reported in Table 1 pointed out that industrial areas have great influence on environmental pollution and increased the incidence of milk contamination by heavy metals. All analyzed milk samples obtained from animals grazing around industrial areas were contaminated by Cu and Al residues and have concentrations higher than that obtained from non-industrial areas. Previously, several studies were carried to detect the concentrations of Cu in camel milk in different areas in the world such as Qassim region of Saudi Arabia [34], Egypt [35], Lybia [36],Yobe State, Nigeria [37] and Kasuwan Shanu market in Maiduguri, Borno State [38], Kazakhstan [39], Kazakhstan [40], Nigeria [41], South Egypt [42], Iran [43] and China [12], they detected Cu at concentrations of 1.61, 1.90, 1.40, 1.34, 2.06 ±0.01, 0.065 ± 0.04, 0.07, 0.161, 0.065±0.02, 0.44 ± 0.03 and 0.248 ±0.55ppm respectively. While [12]detected Al in camel milk at concentration of 0.45± 0.2ppm.

**Table 1:** Concentrations of Cu and Al (ppm) in raw milk samples collected from she-camel grazing in industrial areas and non-industrial areas

**Heavy metals**	**Number of samples**	**Group A (*n*=15)**		**Group B (*n*=15)**		**Mean ± SD of group A**	**Mean ± SD of group B**
		Min	Max	Min	Max		
**Copper**	30	0.02± 0.04 ^a^	0.09± 0.01 ^b^	0.04± 0.03 ^c^	4.38± 0.02 ^d^	0.05 ± 0.04 ^c^	1.43 ± 0.71 ^e^
**Aluminum**	30	ND	0.04± 0.01 ^a^	2.95± 0.04 ^b^	72.49± 0.03 ^c^	0.02 ± 0.01^d^	27.80 ± 1.48 ^e^

Data are mean ± SD (*n* = 30), rows with different letters of superscripts (a, b, c, d and e) differ significantly (*p* < 0.05). Min refers to Minimum, Max refers to Maximum, ND refers to not detected.

The high stability, carcinogenicity, toxicity, and frequent presence of heavy metals in milk have prompted World Health Organization [44] to set up maximum residual limits (MRLs) for Cu (0.1 ppm) and Al (0.05ppm) in raw milk to exclude the possible human toxicity.

In the present study, concentrations of Cu and Al residues were determined in all tested milk samples and compered with MRL of WHO. The data reported in Table 2 pointed out that concentrations of Cu and Al in milk samples of the group A not exceeded the MRL, while in the group B, 60% and 100% of tested milk samples were higher than MRL set for Cu and Al, respectively. High concentrations of Cu and Al in milk samples may be due to environmental pollution in industrial areas.

**Table 2:** Comparing concentrations of Cu and Al in raw camel milk samples with the MRL set by WHO (2007)

**Heavy metals**	**MRL (ppm)**	**Number of examined samples**	**Samples exceeded MRL**			
			**Group A (*n*=15)**		**Group B (*n*=15)**	
			No.	%	No.	%
**Copper**	0.1	30	0	0	9	60
**Aluminum**	0.05	30	0	0	15	100

MRL refers to maximum residual limit

Other factors may enhance heavy metal accumulation in milk such as utensils, especially that made from aluminum and used for milk storage or boiling [14]. Milk is usually boiled for destruction of the microorganisms that contaminate the milk. The effect of milk boiling in different cooking vats is less investigated. Therefore, the effect of boiling in stainless-steel and aluminum containers was investigated in the present study. The data reported in Fig. 2 pointed out that there was no significant difference concerning the Cu levels among raw milk, milk boiled in aluminium pan and milk boiled in a steel pan (*P* > 0.05). While significant difference was found concerning Al level among raw milk, milk boiled in aluminium pan and milk boiled in stainless-steel pan (*P* < 0.05).

Boiling of milk in aluminum containers significantly increased Al levels (*P*< 0.05), which may be due to leaching of Al from containers to the milk causing significant increase in Al load, while boiling in stainless-steel containers reduced the level of both Al and Cu but in small percentage. Almost, similar results were reported by [14, 19]. In contrast to our results, other studies reported that increase in Al levels due to migration from Al containers during heat treatment of milk was relatively low [45, 46]. Low-quality tools usually used for milk boiling, such as Al vats, Therefore, this creates accidental leaching of Al from vats to milk [33].

Regarding the skimming process, data reported in Fig. 3 revealed that the Cu and Al levels were significantly lower in skimmed milk than cream and raw milk (*P* < 0.05). During cream separation, small amount of Cu and Al escaped into the skim milk while greater amount were recovered in the cream. This may be due to heavy metals have an ability to attach to membrane lipoproteins in fat globules and accumulate in fat layer . Our results agreed with the results reported by [33].

With reference to the effect of fermentation on Cu and Al levels in milk samples, data in Table 3 explained that milk fermentation by probiotic bacteria has non-significant effect on the levels of Cu and Al (*P* > 0.05).Almost similar results were reported by [33].

**Table 3:** Effects of fermentation on Cu and Al residue levels in milk samples

**Heavy metals**	**Original sample** **Mean ± SD**	**Fermented milk** **Mean± SD**	***P*-value** **Mannwhitney U test**
**Copper**	1.43 ± 0.71	1.45± .68	0.740
**Aluminum**	27.80 ± 1.48	28.08±1.64	0.604

By concerning other technological process that used by other researchers to reduce heavy metals concentration in milk, the infrared-assisted microwave application may be capable of reducing heavy metals such as Al and Ni concentrations in milk [15]. Also using IMAC HP resin at different conditions is suitable for adsorption of Cu ions from milk and help in its removal [47].

Regarding estrogen and progesterone evaluation, data reported in Table 4 pointed out that grazing of dromedary she camels around industrial areas induce significant reduction in ovarian function showed by reduction of estrogen and progesterone levels and there was a significant negative correlation between heavy metals (Cu and Al) and ovarian hormones levels which reported in Table 5. This may be due to that heavy metals cause disturbances in the reproductive hormones synthesis; Cu reduces mitochondrial activity, damages ovaries, induces apoptosis of cumulus cells and causes abnormalities of sperm [48].

**Table 4:** Estrogen and progesterone concentration in serum of she-camel grazing on industrial and non-industrial areas

**Hormones**	**Number of samples**	**Group A (*n*=15)**		**Group B (*n*=15)**		**Mean ± SD of group A**	**Mean ± SD of group B**
		Min	Max	Min	Max		
**Estrogen (pg/ml)**	30	69± 0.04 ^a^	760± 0.01 ^b^	62.35 ± 0.21^c^	638.46± 0.71^d^	263±0.22 ^e^	336 ± 28.11^f^
**Progesterone (ng/ml)**	30	1.9± 0.14 ^a^	3.3± 0.01^b^	2.95± 0.04 ^c^	72.49± 0.03 ^d^	2.5±0.15 ^e^	2.34 ± 0.51 ^f^

Data are mean ± SD (*n* = 30), rows with different letters of superscripts (a, b, c, d, e and f) differ significantly (*p* < 0.05).Min refers to Minimum, Max refers to Maximum

**Table 5:** Correlation between estrogen and progesterone levels and Cu and Al levels

**Hormones**	**Estrogen**		**Progesterone**	
	r	p	r	p
**Copper**	-0.999	<0.0001	-0.999	<0.0001
**Aluminum**	-0.996	<0.0001	-0.996	<0.0001

There are several categories of contaminants such as industrial chemicals, heavy metals, persistent organic pollutants (POPs), natural toxins, endocrine disruptors, and pesticides [49, 50]. Another name for endocrine-disrupting chemicals is endocrine-disrupting compounds (EDCs). Endocrine disruption" describes the disruption of the hormone system caused by an external agent [51]. The U.S. Environment Protection Agency (EPA) defines an exogenous drug as "any substance that interferes with the body's natural blood-borne hormones-which are responsible for disrupting homeostasis, reproduction, and development-and their synthesis, transport, secretion, binding action, metabolism or elimination." Endocrine disrupting chemicals (EDCs) have a remarkable effect on nuclear receptors (NRs)-regulated reproductive processes, specifically on estrogen receptors (ERs) and androgen receptors via receptor-mediated signal transduction. More precisely, these substances change the production and breakdown of hormones via binding to endocrine receptors. It has been determined that the endocrine disruptors are extremely diverse in nature [52]. By activating, inhibiting, and changing hormonal processes, the chemical substances lead to many clinical consequences, such as infertility, neurodegenerative illnesses and thyroid dysfunctions [52]. This may give explanation to the potential mechanisms by which heavy metal exposure alters ovarian hormone levels and secretion.

The physiological status of camel raised under traditional conditions had more impact on biochemical and hormonal rather than hematological indices [53]. The pattern of reproductive hormones secretion has been reported in different animal species such as sheep, goat, mare, buffalo, cattle, and pig but is less well understood and limited in the camel. Rhythmic secretion of ovarian hormones has a marked correlation with receptivity and sexual behavior of the male by females in other species of livestock [54].

## Conclusions

In conclusion, the findings of this study indicate that camels who are allowed to graze freely in industrial areas may accumulate heavy metals in their organs and tissues, which are then excreted in their milk. Presence of high levels of Cu and Al in milk samples indicate that environmental pollution with heavy metals poses a danger to human and animal health. Regular and routine monitoring of heavy metals concentration in milk and other foods especially in industrial areas is necessary to ensure safety and quality of such products to consumers. Heating of milk in Al pan should be prohibited as it increases Al levels in milk. Fermentation has no significant effect on reduction of Cu and Al levels in milk. Skimming have significant effect especially in the skimmed portion while the cream layer has the greater part of heavy metals residues. Investigation of ovarian hormones (estrogen and progesterone) revealed presence of a signification reduction in the levels of these hormones. There is a negative correlation between Cu and Al levels and ovarian hormones (estrogen and progesterone) levels. Many studies are required to determine the extent of the harmful effects of Cu and Al intoxication on reproductive activity in she-camel. Also farther studies include assessments of samples from the environment as soil samples, air, water and feed stuffs are required, and a parallel serum toxicological analysis would provide some data.

## Data Availability

The data presented in this study are not deposited in an official repository. Data are available within the article and from the corresponding author upon reasonable request.
